# Cell-derived vesicle-modified biomaterials for tissue repair and regeneration

**DOI:** 10.1016/j.mtbio.2026.103263

**Published:** 2026-05-23

**Authors:** Wanli Song, Junmiao Xue, Pengfei Jia, Yongzhi Xu, Hao Jiang, Zhiyu Yuan, Tianyi Zhang, Wenping Zhang, Jingyang Zhao, Xiaohui Qiu, Qihui Zhou

**Affiliations:** a NHC Key Laboratory of Cardiopulmonary Rehabilitation and Functional Recovery (University of Health and Rehabilitation Sciences), Shandong Key Laboratory of Neurorehabilitation, Shandong Engineering Research Center for Tissue Rehabilitation Materials and Devices, Qingdao Key Laboratory of Smart Rehabilitation Material, School of Rehabilitation Sciences and Engineering, University of Health and Rehabilitation Sciences, Qingdao, 266113, China; b Department of Dental Materials & Dental Medical Devices Testing Center, Peking University School and Hospital of Stomatology, Beijing, 100081, China; c School of Stomatology, Qingdao University, Qingdao, 266003, China; d Qingdao Stomatological Hospital Affiliated to Qingdao University, Qingdao, 266001, China; e Department of Stomatology, Qingdao Women and Children's Hospital, Qingdao, 266011, China

**Keywords:** Tissue repair and regeneration, Bioactive materials, Cell-derived vesicles, Cell membrane vesicles, Exosomes

## Abstract

In recent years, cell-derived vesicle-modified biomaterials (CDVMBs) have been considered a promising strategy to overcome the limitations of traditional biomaterials in tissue repair and regeneration. By combining biomaterial carriers with cell-derived vesicles, CDVMBs integrate the functional advantages of the carriers, including support for cell adhesion, colonization, proliferation, and functionalization, while also incorporating the bioactive properties of cell-derived vesicles. In this review, the term “cell-derived vesicles” refers to two distinct bioinspired components used for carrier modification: cell membrane vesicles, which mainly retain membrane-associated receptors and interfacial biological functions, and exosomes, which are nanosized extracellular vesicles enriched in bioactive cargos such as proteins and nucleic acids. Accordingly, CDVMBs can mimic either the surface biological properties of source cells or the signaling functions mediated by exosomal cargos, thereby promoting interactions with damaged tissues and stimulating tissue regeneration. Based on the biomaterial biomimetic strategy and vesicle source, CDVMBs are classified into cell membrane-camouflaged biomaterials (CMCBs) and exosome-modified biomaterials (EMBs). This review summarizes their engineering strategies, biological mechanisms, and versatile applications for tissue repair, and further discusses the current challenges and future perspectives for clinical translation. Taken together, the integration of biomaterial carriers with cell-derived vesicles establishes a versatile bioinspired framework for engineering regenerative microenvironments and advancing tissue repair and regeneration.

## Graphical abstract

**Figure:**
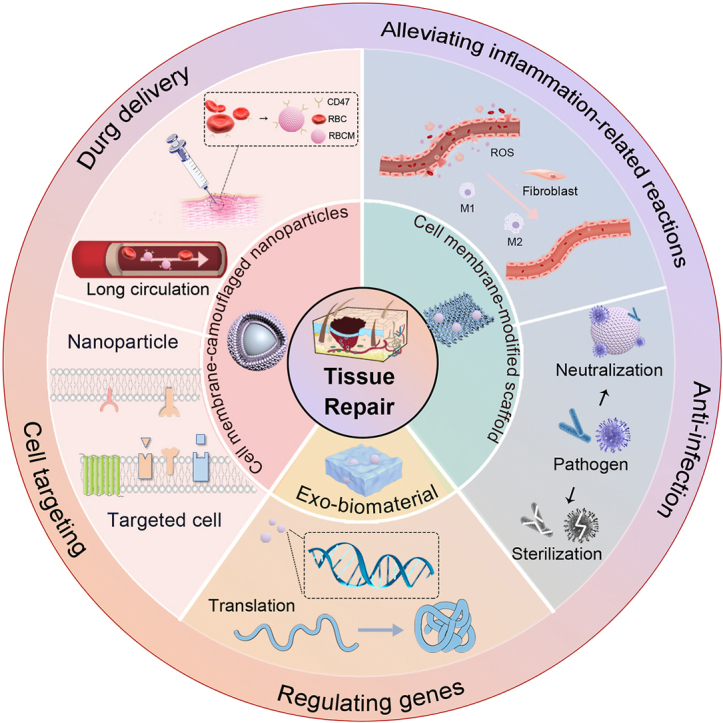

## 1 Introduction

Tissue repair and regeneration is a complex biological process that aims to restore the structure and function of damaged tissues through coordinated inflammatory responses, cell proliferation, and extracellular matrix (ECM) remodeling [1]. Although these events are often described as inflammation, proliferation, and remodeling, they are highly overlapping and dynamically regulated rather than strictly separated [[2], [3], [4], [5], [6]]. In many pathological conditions, persistent infection, excessive inflammation, large tissue defects, and fibrotic healing impair this process and result in incomplete functional recovery [[7], [8], [9], [10], [11], [12]]. The intrinsic regenerative capacity of damaged tissues is often insufficient to overcome these barriers, especially in complex or large-scale defects, underscoring the need for external therapeutic interventions that provide both structural support and biological regulation. Therefore, tissue engineering strategies that integrate biomaterial support with biological regulation have attracted increasing attention for improving tissue repair and regeneration.

Traditional clinical strategies, such as autografts and allografts, have been widely used for bone defect repair, wound closure, and cartilage regeneration, but their clinical application remains limited by donor shortage, surgical trauma, infection risk, and prolonged treatment time [[13], [14], [15]]. A variety of emerging biomaterials, including nanoparticles (NPs), microneedles, hydrogels, electrospun nanofibers, and three-dimensional (3-D) scaffolds have attracted extensive attention in tissue repair and regeneration due to the excellent (bio)physicochemical properties, flexible modification, and cargo-loading capacity [[16], [17], [18], [19], [20], [21], [22], [23], [24]]. However, biomaterials are often recognized as foreign substances after implantation, which may trigger immune activation and foreign body reactions (FBRs) [25], disrupt interactions among immune cells, endothelial cells, and endogenous stem cells, and ultimately compromise tissue repair [26]. These limitations have driven the development of biomimetic biomaterials with improved biocompatibility and biological functionality [27].

Among these strategies, cell-derived vesicle-modified biomaterials (CDVMBs) have emerged as a promising class of bioinspired materials for tissue repair and regeneration [[28], [29], [30]]. In this review, the term “cell-derived vesicles” (CDVs) refers to two distinct bioinspired components used for biomaterial modification: cell membrane vesicles and exosomes. Cell membrane vesicles are typically generated by the physical disruption and reassembly of cellular membranes and primarily preserve membrane-associated lipids, receptors, adhesion molecules, and other surface proteins inherited from the source cells. By contrast, exosomes are naturally secreted nanosized extracellular vesicles, generally 30–150 nm in size, enriched in proteins, lipids, nucleic acids, and other bioactive cargos involved in intercellular communication. Therefore, although both fall under the broader category of cell-derived bioinspired vesicles, they differ in origin, size, composition, and biological function. Cell membrane-derived vesicles primarily confer biomimetic interfacial properties, such as immune evasion, lesion targeting, and toxin neutralization. In contrast, exosomes primarily deliver bioactive cargos and regulate signaling pathways related to inflammation, proliferation, angiogenesis, and tissue remodeling [[31], [32], [33]]. By preserving the admirable physicochemical properties of the carriers, cell membrane vesicles further endow CDVMBs with unparalleled biomimetic performance [34]. Accordingly, CDVMBs can be broadly divided into two categories: cell membrane-camouflaged biomaterials (CMCBs) and exosome-modified biomaterials (EMBs). CMCBs primarily use membrane-derived vesicles to endow biomaterials with cell-like surface properties. In contrast, EMBs mainly use biomaterials as carriers to improve the retention, protection, and local delivery of exosomes at injury sites. CMCBs and EMBs share the common goal of improving the bioactivity and therapeutic efficacy of biomaterials, but they differ in design logic and biological emphasis. CMCBs mainly focus on reconstructing a biomimetic interface through membrane-associated receptors and functional proteins, thereby enhancing targeting capability, prolonging circulation, reducing immune clearance, and modulating local inflammatory responses [35,36]. EMBs, in contrast, mainly focus on harnessing exosome-mediated cargo delivery and intercellular communication to regulate cell behavior and promote tissue regeneration [[37], [38], [39]]. By clarifying the distinction between CMCBs and EMBs and highlighting their respective advantages in regenerative applications, this review aims to provide a useful framework for the rational design of next-generation biomaterials for tissue repair and regeneration.

**Fig. 1:**
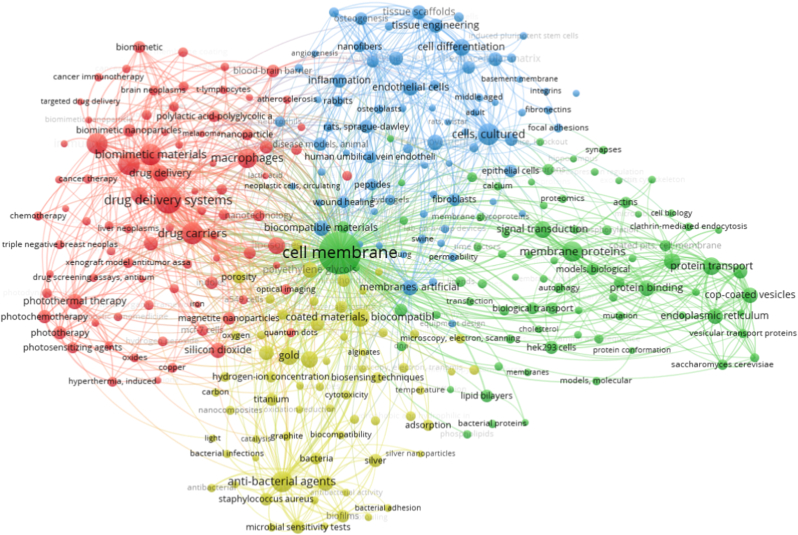
The analysis of keyword co-occurrences on tissue repair and regeneration, cell-derived vesicle-modified biomaterials, cell membrane-camouflaged nanoparticles, cell membrane-modified biological scaffolds, and exosomes.

In this review, we systematically summarize the recent progress of CDVMBs for tissue repair and regeneration, with particular emphasis on their design principles, representative categories, and biological roles in regulating inflammation, cell proliferation, and ECM remodeling. To contextualize the research landscape of cell-derived vesicle-modified biomaterials (CDVMBs), we employed VOSviewer software to conduct a bibliometric keyword co-occurrence analysis (Fig. 1), which shows that CDVMB-related research is mainly distributed across four thematic domains: biomimetic drug delivery, regenerative medicine, membrane biology, and material-functionalization research. Although tissue repair and regeneration has emerged as a distinct branch, its integration with membrane engineering and vesicle biology remains relatively limited, highlighting the need for more mechanism-driven and tissue-specific design of CDVMBs. Compared with existing reviews that separately focus on cell membrane-vesicle modified or exosome-modified therapies, this review establishes a unified, materials-oriented engineering framework to systematically integrate CMCBs and EMBs, with a unique focus on spatiotemporal regulatory mechanisms and translational challenges in tissue repair, thereby providing a distinct and comprehensive perspective for rational design of next-generation CDVMBs. By integrating the structural support of biomaterials with the biological functions of cell-derived vesicular components, CDVMBs provide a versatile platform for overcoming the limitations of conventional biomaterials in tissue repair [[40], [41], [42], [43], [44]].

## 2 Biological basis and bioengineering strategies of CDVMBs

CDVMBs are a distinctive class of bioinspired materials that combine the structural and physicochemical advantages of biomaterial carriers with the biological functions of vesicular components derived from living cells. For tissue repair and regeneration, this combination addresses two long-standing limitations of conventional biomaterials: low biological specificity and poor integration with the dynamic microenvironment of injured tissues. Thus, the key question is not simply which vesicle type to use, but how to select and integrate different vesicular components to match the biological barriers and regenerative demands of a given lesion. This section first clarifies the biological basis of the vesicular components used in CDVMBs and then discusses the major bioengineering strategies for incorporating these components into biomaterial systems (Table 1) .

**Table 1:** Classification of cell-derived vesicles in this review.

Types	Extraction methods	Sources	Molecules/Cargos	Functions
Cell membrane vesicles	Membrane extraction, purification, and reassembly	Red blood cells	CD47 (engages SIRPα to reduce phagocytic clearance)	Immune evasion; prolonged circulation
		Platelets	P-selectin, glycoprotein receptors	Damaged-vessel recognition; subendothelial interaction; vascular injury and thrombo-inflammatory lesions
		Neutrophils	CD11b/CD18, LFA-1, Mac-1	Inflammatory homing; adhesion to ICAM-1; transendothelial migration in inflamed tissues
		Macrophages	CCR2-CCL2 axis, integrin-VCAM/ICAM axis	Lesion tropism; immune communication; inflammatory scavenging in cytokine-rich microenvironments
Exosome	Naturally secreted (30-150 nm)	Mesenchymal stem cells	Immunomodulatory, angiogenic, anti-apoptotic, osteogenic/chondrogenic signaling molecules	Regenerative medicine
		Macrophages	Factors that influence macrophage polarization, cytokine balance, and stromal-cell behavior	Inflammation-associated repair
		Endothelial cells	Angiogenic support molecules	Endothelial repair
		Neural/Schwann cells	Neuroprotective and axonal regeneration factors	Neuroprotection; axonal regeneration
		Platelets	Hemostatic and regenerative signaling molecules	Vascularized and wound-healing contexts

**Table 2:** Summary of representative CMCBs for tissue repair and regeneration.

Cell Source	Core material	Engineering function	Mechanism	Design value	Ref
RBC membrane	Methacrylated alginate with RBC membrane coated nanoparticles	Anti-FBR interface regulation	Reduces early host recognition and inflammatory infiltration, improving scaffold-host compatibility	Better as an interface-regulating strategy than active targeting	[24]
Platelet-macrophage hybrid membrane	Nanocomplex loaded with siSav1	Dual lesion targeting	Integrates vascular injury recognition and inflammatory homing for myocardial delivery	Valuable when neither vascular nor inflammatory targeting alone is sufficient	[45]
Macrophage membrane	Magnetic nanoclusters containing AP and ST	Inflammatory plaque targeting and oxidative damage reduction	Leukocyte-like affinity improves lesion accumulation in atherosclerosis; reduces lipid and DNA damage	Suitable for chronic vascular inflammatory lesions needing both targeting and local intervention	[46]
	Ca_3_(PO_4_)_2_ with TiO_2_	Anti-infective targeting and inflammatory interception	Combines antibacterial activity and toxin neutralization	Suitable for infected bone defects requiring simultaneous bacterial control	[47]
	PLGA nanoparticles loaded with rapamycin	Plaque targeting and local anti-inflammatory delivery	Enhances accumulation in atherosclerotic lesions and improves local drug release	Useful when poor localization of anti-inflammatory therapy is the dominant challenge	[22]
	MnO_2_ nanospheres loaded with fingolimod (FTY)	Inflammatory lesion targeting and local immunoregulation	Combines ROS modulation and macrophage phenotype regulation to support neuronal repair in ischemic lesions	Suitable for acute inflammatory injuries needing both targeting and immune reprogramming	[48]
	MMNPs loaded with miR199a-3p	Antifibrotic cargo delivery	Membrane-mediated lesion interaction with miRNA regulation of fibrosis and inflammation after MI	Typical strategy using membrane camouflage to enhance delivery of gene-regulatory cargos	[49]
PRP membrane	PLPMH hydrogel	Anti-inflammatory and pro-regenerative microenvironment construction	Provides bioactive microenvironment that supports M2 immune balance and cartilage regeneration	Useful when inflammation control and pro-regenerative signaling are both required	[50]
	PRP coated PCL scaffold	Pro-regenerative biointerface enhancement	Maintains membrane-associated growth factors; improves cell apposition, proliferation, and angiogenesis	More valuable as a bioactive scaffold interface than a targeting system	[51]
Neutrophil membrane	IRF-5SiRNA with M@pMn nanozyme (Mn_3_O_4_ based)	Inflammatory targeting plus oxidative and immune regulation	ROS scavenging with macrophage phenotype reversal to reduce scarring and support recovery in SCI	Useful for lesions where inflammatory targeting and secondary injury control are both important	[52]
Beta-cell membrane	CM fibers on polymeric nanofibers	Cell-instructive scaffold functionalization	Provides topographic cues and membrane-derived biointerfaces to improve cell survival and bioactivity	Extends membrane camouflage from nanoparticles to scaffolds	[23]
Fibroblast membrane	Electrospun scaffold	Cell-specific interfacial signaling	Mimics native fibroblast microenvironment	Actively instruct resident-cell behavior beyond adhesion	[53]

**Table 3:** Summary of representative EMBs for tissue repair and regeneration.

Exosome source	Carrier material	Engineering function	Mechanism	Design value	Ref
Adipose derived MSC exosomes	Type I collagen with platelet rich plasma scaffold	Local retention and scaffold assisted regenerative stimulation	Enhances fibroblast proliferation and wound closure by combining exosomal trophic signaling with bioactive scaffold matrix	More suitable for wound beds requiring both exosomal signaling and provisional matrix support	[54]
Adipose derived MSC exosomes	ECM derived hydrogel	Sustained release and local exosome preservation	Promotes HUVEC proliferation and supports vascularized chronic wound repair through prolonged exosome retention	Represents a typical EMB strategy where the biomaterial mainly improves retention and release behavior rather than serving as a passive carrier	[55]
Engineered neutrophil derived exosomes (eNABs)	HAL loaded MSNHAL based engineered platform	Engineered multifunctional exosomes delivery	Combines exosome engineering with inflammatory homing and bilirubin related immunoregulation to reduce infarct size after myocardial infarction	Useful when both lesion targeting ability and engineered exosomal function are needed	[56]
Bone marrow MSC derived exosomes	Minimally invasive spray forming an *in situ* fibrin patch	Patch assisted local retention and delivery	Improves exosome retention and uptake in infarcted heart, thereby reducing cardiomyocyte apoptosis and infarct size	Illustrates the value of EMBs in highly dynamic tissues where free exosomes are otherwise rapidly lost	[57]
Human MSC derived exosomes	Adhesive hydrogel	Adhesive localization in complex lesions	Reduces local inflammation and supports functional spinal cord repair by immobilizing exosomes within an injury matched hydrogel	Particularly valuable in anatomically complex lesions where free exosomes show poor retention	[58]

### 2.1 Cell membrane vesicles and exosomes: definitions, sources, and functional roles

In this review, CDVMBs refer to biomaterial systems functionalized with vesicular components derived from eukaryotic cells, including cell membrane vesicles and exosomes or exosome-enriched small EVs. Since many studies use the term “exosome” without fully demonstrating vesicle biogenesis, this review follows the terminology adopted in the original reports [59].

Although cell membrane vesicles and exosomes both confer biological functions on materials, they differ fundamentally in generation, composition, and intended role after incorporation into a biomaterial platform. Cell membrane vesicles are typically obtained through membrane isolation, purification, and reassembly. Their main value lies in preserving membrane-associated lipids, receptors, glycans, adhesion molecules, and other surface proteins inherited from source cells. In practice, they primarily serve to build a biomimetic interface. Exosomes, in contrast, are naturally secreted nanoscale vesicles enriched in proteins, lipids, mRNAs, microRNAs, and other signaling molecules involved in intercellular communication. Their principal value lies in their cargo-mediated biological activity, particularly their ability to modulate the behavior of recipient cells. The distinction between CMCBs and EMBs therefore reflects not only a difference in vesicle source, but also a difference in design logic: CMCBs are primarily used to reconstruct biologically active surfaces, whereas EMBs are mainly used to preserve and deliver vesicle-borne regulatory signals.

The biological relevance of CDVMBs is highly source-dependent. For CMCBs, different cell types express distinct membrane receptors, which are consequently suited to different therapeutic scenarios [60,61]. Red blood cell membranes are typically selected when immune evasion and prolonged circulation are prioritized, largely because membrane-associated CD47 can engage SIRPα and reduce phagocytic clearance. Platelet membranes are better suited for vascular injury and thrombo-inflammatory lesions, as platelet-derived interfaces retain adhesion-related molecules, such as P-selectin and glycoprotein receptors, that naturally participate in damaged-vessel recognition and subendothelial interaction. Neutrophil membranes are particularly relevant to acute inflammatory lesions because they preserve the inflammatory homing machinery. Adhesion molecules such as CD11b/CD18, LFA-1, and Mac-1 can interact with ICAM-1 and related endothelial ligands, thereby facilitating adhesion, retention, and transendothelial migration in inflamed tissues. Macrophage membranes are particularly attractive in cytokine-rich inflammatory microenvironments, where receptor-ligand axes such as CCR2-CCL2, integrin-VCAM/ICAM, and other inflammation-associated interactions may support lesion tropism, immune communication, and inflammatory scavenging. Taken together, these examples indicate that membrane source selection should be guided not by generic “targeting ability” but by the match between inherited membrane receptors and the dominant biological barrier of a given tissue defect [62].

A similar principle applies to EMBs; however, the decisive factor in this context is less the surface identity of the vesicle and more the functional repertoire of its encapsulated cargo [63]. Mesenchymal stem cell-derived exosomes are extensively used in regenerative medicine research. They exhibit immunomodulatory, angiogenic, anti-apoptotic, and osteogenic or chondrogenic activities. Macrophage-derived exosomes are more closely associated with inflammation-related repair, as they can influence macrophage polarization, cytokine balance, and stromal-cell behavior. Endothelial-cell-derived exosomes are particularly relevant to vascular repair and angiogenesis, whereas neural- or Schwann-cell-derived exosomes are better suited for neuroprotection and axonal regeneration. Platelet-derived exosomes occupy an intermediate niche bridging hemostatic responses and regenerative signaling cascades, thus holding particular promise for vascularized tissue regeneration and wound-healing applications. Collectively, for both membrane vesicles and exosomes, source selection should be guided by the primary biological task at the injury site rather than by source availability alone.

Taken together, cell membrane vesicles and exosomes should be regarded as two distinct but complementary biological building blocks for biomaterial design. One mainly contributes interfacial recognition and biomimetic surface function; the other mainly contributes signal transfer and cargo-mediated regulation. This distinction provides the biological basis for the engineering strategies discussed below.

### 2.2 Bioengineering strategies for integrating vesicles with biomaterials

**Fig. 2:**
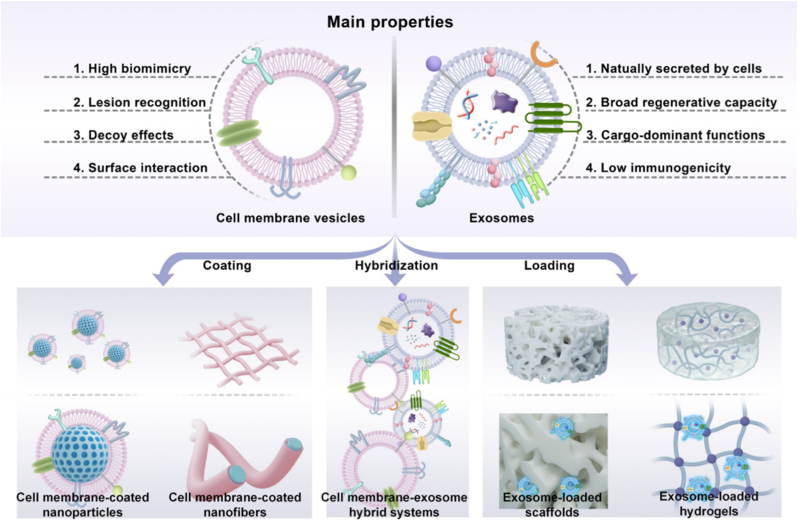
Properties and design strategies of cell membrane vesicles and exosomes for biomaterials in tissue repair and regeneration.

From the standpoint of biomaterials engineering, CDVMBs are best understood not by vesicle source alone, but by how vesicular components are integrated with material platforms to achieve specific regenerative functions. Three major design strategies can be identified (Fig. 2): **1)** membrane coating or camouflage, **2)** vesicle loading and immobilization, and **3)** hybrid vesicle–material construction. These strategies are not merely different fabrication methods but distinct ways of coupling vesicle-derived functions with material architecture to address distinct problems in tissue repair.

The first strategy is membrane coating or camouflage, in which cell membrane vesicles are assembled onto the surfaces of nanoparticles, nanofibers, and scaffolds to construct a biomimetic interface. The primary engineering objective is to modulate host-mediated recognition of the material. The therapeutic efficacy of such systems hinges on the functional preservation and correct orientation of membrane-associated receptors, adhesion molecules, and lipid bilayer characteristics post-coating. Common fabrication approaches include membrane extraction followed by extrusion, sonication, electroporation, microfluidic fusion, or interfacial self-assembly with the core platform. Given that the biological performance of these systems depends on the presentation of membrane-derived cues at the surface, several physicochemical parameters are particularly significant, including coating integrity, membrane orientation, membrane fluidity, receptor exposure, and colloidal stability. Intrinsic material properties, such as size, shape, elasticity, surface roughness, and charge, can influence whether the membrane wraps efficiently and remains functionally intact. This strategy is particularly useful when the dominant challenge is interface-related, such as immune clearance, inflammatory lesion recognition, vascular injury targeting, toxin neutralization, or modulation of early host–material interactions.

The second strategy is vesicle loading and immobilization, in which exosomes are incorporated into biomaterials to improve their retention, protection, and local bioavailability. In this case, the exosome is not primarily used as a coating layer, but as a functional payload whose persistence and release need to be controlled. Depending on the material system, exosomes may be physically entrapped during gelation, adsorbed after fabrication, immobilized through affinity interactions, or anchored by covalent or coordination chemistry. These modes of incorporation are not interchangeable, as they determine whether exosomes are released rapidly, retained for sustained delivery, or presented locally over time. Accordingly, the key design variables differ from those in membrane coating systems: here, the most relevant parameters are porosity, crosslink density, swelling behavior, degradation kinetics, rheology, and surface chemistry, all of which govern loading efficiency, burst release, long-term retention, and preservation of cargo activity. This strategy is generally preferred when the main therapeutic obstacle is poor local persistence of exosomes or insufficient sustained biological signaling, as in chronic wounds, poorly vascularized defects, or tissues that require prolonged trophic support. Within this second strategy, scaffold-based systems are especially important for regenerative applications. Hydrogels, electrospun membranes, porous scaffolds, microneedles, and adhesive patches can all serve as local vesicle depots, but their roles extend beyond exosome storage. In many repair settings, they also provide mechanical support, improve lesion matching, and help coordinate vesicle release with the evolving phases of tissue repair. For example, in chronic skin wounds, a hydrogel may reduce burst loss and maintain a moist microenvironment while continuously releasing exosomal cues. In bone defects, a porous scaffold may provide structural guidance while enabling gradual signal presentation within a three-dimensional regenerative niche. In myocardial or spinal cord injury, patch- or hydrogel-based systems may improve exosome retention in anatomically complex, highly dynamic tissues where direct injection alone is inefficient.

The third strategy is hybrid vesicle-material construction, in which membrane-derived materials, exosomes, liposomes, polymers, or inorganic nanomaterials are integrated into a single multifunctional platform. Hybridization is attractive when neither membrane camouflage nor exosome loading alone is sufficient to meet the therapeutic demands of a given lesion. For instance, membrane-derived components may provide lesion recognition or immune camouflage, while synthetic modules contribute imaging capability, structural robustness, responsiveness, or staged release. Likewise, an exosome-based system may be combined with a synthetic or membrane-derived module to improve localization, durability, or multi-step therapeutic action. From an engineering perspective, hybrid systems are particularly relevant in complex lesions that require simultaneous targeting, retention, and controlled biological signaling. Their main advantage lies in functional complementarity. Their main drawback is that increased complexity often makes reproducibility, quality control, and mechanistic interpretation more difficult.

Importantly, these three strategies can be implemented across multiple material platforms, including nanoparticles, microparticles, hydrogels, electrospun membranes, porous scaffolds, microneedles, and patches. The choice of platform should depend on both the biological target and the anatomical context of the defect. Nanoscale platforms are generally advantageous for circulation, deep lesion penetration, or multifunctional delivery. By contrast, scaffold- or hydrogel-based systems are more suitable when local retention, tissue matching, and structural support are priorities. In practical terms, the selection of a design strategy should be guided by three questions. First, what is the dominant biological barrier in the target tissue: immune clearance, inflammatory lesion recognition, poor local vesicle persistence, or insufficient trophic signaling? Second, is the desired biological action mainly surface-mediated or cargo-mediated, and does it need to be an immediate onset or long-term sustained action? Third, which material architecture is best suited to preserve the relevant vesicle function under the mechanical and biological conditions of the lesion? In general, membrane coating is more appropriate when biomimetic interface control is the priority, vesicle loading is more appropriate when sustained exosomal signaling is required, and hybrid systems are more suitable when complex lesions demand multiple therapeutic functions at once.

## 3 Cell membrane-modified biomaterials (CMCBs) for tissue repair and regeneration

**Fig. 3:**
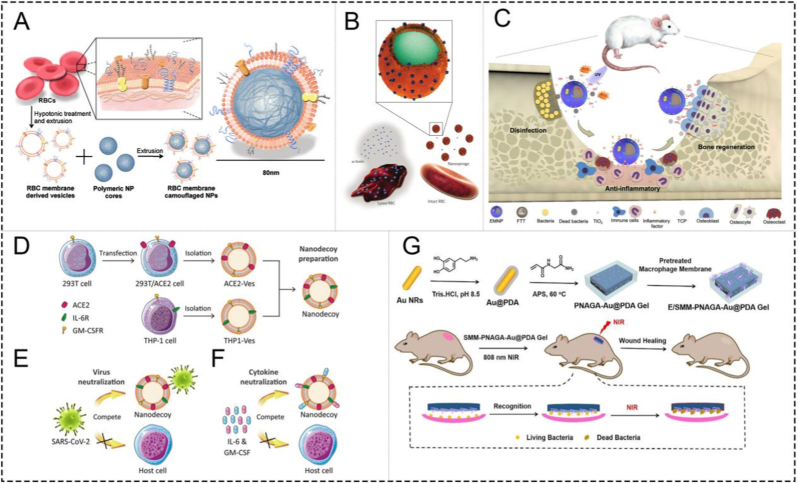
**(A)** Schematics of the preparation process of the RBC-membrane-coated PLGA nanoparticles [64]. **(B)** Schematic illustration of RBC nanosponges (denoted “RBC-NPs”) protect host cells from bacterial toxin attacks [65]. **(C)** Schematic diagrams of the preparation and application of membrane-coated nanoparticles by electroporation (EMNPs) [47]. **(D)** Preparation of nanodecoys by fusing cellular membrane nanovesicles derived from genetically edited 293T/ACE2 and THP-1 cells. The nanodecoys, displaying abundant ACE2 and cytokine receptors, compete with host cells and protect them from COVID-19 by neutralizing [66]. **(E)** SARS-CoV-2 and **(F)** inflammatory cytokines, such as IL-6 [66]. **(G)** Schematic illustration of the fabrication of a high-strength and tough E/SMM-PNAGA-Au@PDA nanocomposite hydrogel with a targeted binding to specific bacteria and efficient antibacterial activity for promoting wound healing [67].

Due to the excellent physicochemical properties but the innate limitations of biomaterials for tissue repair and regeneration, cell membrane-coated biomaterials, commonly referred to as CMCBs, have been extensively investigated in clinical studies, which can be classified as CMCNPs and CMMBSs based on different biomaterials. Multifunctional nanoplatforms with natural cell membrane camouflage typically involve three main steps to achieve self-assembly: (1) separation of the cell membrane by means of hypotonic treatment, freeze-thaw cycle, or differential centrifugation; (2) synthesis of the core biomaterial of nanoplatforms; (3) final assembly of the cell membrane vesicles and biomaterial NPs together to form core-shell nanostructures through co-extrusion, sonication, microfluidic electroporation, spontaneous formation by electrostatic attractions, and polymerization *in situ*, *etc* [61]. In addition, the cell membrane can be attached to the scaffold by stirring, providing the possibility of chemical bonding of the active groups on the surface of the cell membrane with the scaffold, such as hydrogel, microneedles, and electrostatic fiber spinning (electrospinning), *etc.* In 2011, Zhang et al. first coated poly lactic-co-glycolic acid (PLGA) with the red blood cell (RBC) membrane to achieve excellent biocompatibility and long circulation time (Fig. 3A) [64]. CD47, a kind of protein molecule on the surface of the erythrocyte membrane, could send the signal "do not eat me", thereby avoiding phagocytosis by macrophages and improving the bioavailability of the drug [68]. While retaining the physicochemical properties of the core biomaterials, they also inherit the surface functional proteins and sponge-like phospholipid bilayer structure of the original cell membranes, making them versatile like natural cells to achieve active-targeting effect at lesion sites, biological toxin neutralization, and ideal biocompatibility. For instance, by mimicking the characteristics of source cells, Zhang et al. used cell membranes to fabricate "cell nanosponges", which is a representative example of a cell-mimicking strategy [65]. With the help of their surface receptors and unique phospholipid bilayer, the spongy-like structure of cell membranes could neutralize various toxins and inflammatory factors as decoys, thereby terminating their impact on the host immune system and inflammatory phase (Fig. 3B).

In recent years, CMCBs have become increasingly popular for regulating inflammation and promoting tissue remodeling, and are accordingly regarded as promising candidates in this field. In terms of inflammation regulation, CMCBs supply the neutralization effect with a sponge-like porous structure by absorbing various toxins and inflammatory cytokines, and achieving targeted drug delivery to pathogens and inflammatory sites by recognition of specific receptors on the cell membrane surface. While for the promotion of tissue remodeling, CMCBs can not only promote cell proliferation and bioactivity, but also reduce scar formation and fibrosis. In this section, the latest advances in CMCBs for regulating inflammation and promoting tissue remodeling will be discussed from different perspectives, as well as detailed research reports.

### 3.1 CMCBs for infection and inflammatory microenvironment modulation

Building on the conceptual and engineering framework outlined above, this section focuses on CMCBs as a major class of CDVMBs for tissue repair and regeneration. Owing to the membrane-associated receptors, adhesion molecules, and biomimetic interfacial properties inherited from source cells, CMCBs can regulate host–material interactions, improve lesion recognition, and enhance therapeutic specificity in complex pathological microenvironments. Functionally, their roles in tissue repair can be broadly reflected in two aspects: the modulation of infection- and inflammation-associated microenvironments, and the promotion of regenerative remodeling and functional restoration. Accordingly, the following discussion is organized around these two dimensions to highlight how membrane camouflage contributes to different stages during tissue repair. The rational regulation of inflammation has been a top priority in tissue repair and regeneration research in recent years [69,70]. Through cell membrane coating of NPs or scaffolds, CMCBs exhibit more satisfactory biocompatibility and longer blood circulation. Overcoming the limitations of traditional immune-modulating drugs, such as drug resistance and poor targeting, CMCBs have attracted considerable attention due to their excellent pathogen clearance, inflammation site targeting, and immunomodulatory capabilities [28]. Not only can CMCBs rely on their core biomaterials to kill pathogens, but also identify inflammatory receptors and achieve precise targeting of inflammatory sites with the help of bionic membrane surfaces. In addition, inflammatory factors could be neutralized, and immune balance could also be regulated, relying on the sponge-like structure of the cell membrane. In this section, the research progress, mechanism, and effectiveness of CMCBs for anti-infection, targeted inflammation, and immune regulation are expounded systematically.

#### 3.1.1 Pathogen interception and anti-infection applications

Infection refers to the local and systemic inflammatory reactions caused by the invasion of bacteria, viruses, fungi, parasites, and other pathogens into the human body, which can lead to tissue defects in severe cases, such as infectious bone defects [71]. Therefore, eliminating infection and managing the inflammatory response are vital during tissue repair and regeneration [72]. Although antibiotics remain a common therapeutic strategy, the increasing emergence of antimicrobial resistance has highlighted the need for alternative anti-infective approaches that can more effectively control infection while supporting tissue healing [[73], [74], [75]]. In recent years, nano-biomaterials with unique (bio)physicochemical properties have shown remarkable anti-infection effects, and various nanobiomaterials have been explored as antibiotic-sparing anti-infective platforms [76,77]. However, their therapeutic performance is often limited by insufficient biocompatibility, poor lesion specificity, and inadequate interaction with complex infectious microenvironments. Cell membrane coating significantly enhances the biocompatibility and prolongs the blood circulation of the above NPs. Meanwhile, NPs inherit the cell membrane receptors to imitate the biological dynamic behavior of the original cell, while also enabling pathogen interception, toxin neutralization, and infection-associated lesion recognition. For example, neutrophil membrane-camouflaged NPs can transmigrate to inflammatory sites, and macrophage membrane-camouflaged NPs can recognize and target PAMPs [78,79]. It is worth mentioning that the main component of the cell membrane, the phospholipid bilayer, can achieve toxins and pathogenic microorganism absorption, acting as a cellular decoy that reduces the binding and subsequent destruction of normal cells, which has been taken into consideration for therapy of sepsis, Zika virus, COVID-19, *etc* [80]. Therefore, compared with conventional antimicrobial nanomaterials, CMCBs are distinguished not simply by their bactericidal activity, but by their ability to couple anti-infective functions with biomimetic lesion interaction.

This membrane-enabled strategy has been explored in several infection-related settings. Severe inflammation and progressive bone destruction are two main characteristics of bone infection, which might induce fractures and sepsis [81]. Significant bone tissue damage and immune response are triggered following the production of matrix enzymes and inflammatory cytokines [82]. Antibacterial therapy and effective anti-inflammatory therapy are usually required for the treatment of bone infections. In addition, degradable bone substitutes are considered to induce bone tissue repair and regeneration. Shi et al. prepared a macrophage membrane-coated biomaterial combining Ca_3_(PO_4_)_2_ as the core of the bone repair scaffold with titanium dioxide (TiO_2_) with strong photocatalytic ability as the antibacterial component. The composite biomaterial in this work achieved active targeting and enhanced antibacterial capability (Fig. 3C) [47]. It is worth noting that the sponge-like structure of the cell membrane, along with its surface receptors, can neutralize bacterial toxins and inflammatory cytokines, preventing further inflammation. In promoting bone repair, CMCBs innovatively combine core bone repair materials with cell membrane biomimetic technology. By modifying traditional antibacterial and scaffold materials with cell membranes, a variety of receptor proteins on the cell membrane achieve targeted effects on bacteria and neutralize toxins, enhancing the value of traditional materials and providing new insights for the design of future bone repair materials. However, the respective contributions of membrane camouflage and the TiO_2_-containing core were not fully distinguished, and the long-term stability of the coated membrane in the mechanically dynamic bone environment remains unclear.

A similar decoy principle has also been applied to viral infection. Given the high lethality of the novel severe acute respiratory syndrome coronavirus 2 (SARS-CoV-2), highly effective protective strategies against ongoing COVID-19 and potential future pandemics remain an intractable challenge [66,67,83]. Rao et al. reported a nanodecoy (Fig. 3D) for COVID-19 using a powerful two-step neutralization approach: neutralizing the virus in the first step (Fig. 3E) and neutralizing the inflammatory cytokines in the second step (Fig. 3F) [66]. The nanodecoys, fusing nanovesicles derived from THP-1 cells and genetically transfected 293T cells, had the same cell-surface antigenic profile as the source cells. As nanodecoys competed with host cells for viral binding, they effectively prevented host cells from being infected by SARS-CoV-2. In addition, nanodecoys could effectively bind and neutralize inflammatory cytokines, depending on the abundance of cytokine receptors on the surface, including IL-6 and granulocyte-macrophage colony-stimulating factor (GM-CSF), and could effectively inhibit immune disorders and lung injury, which was promising for lung tissue repair and regeneration. However, the reliance on engineered source cells and membrane fusion may complicate large-scale production and standardization, and its applicability to viral variants and repeated administration still requires further validation.

In addition to nanoparticle systems, membrane camouflage has also been integrated into multifunctional wound dressings and hydrogels for infection-associated tissue repair. [84]. Li et al. developed a novel multifunctional, tough, antibacterial nanocomposite hydrogel that effectively recognized and killed target bacteria [67]. In brief, the hydrogel was prepared by one-pot polymerization of hydrogen-bonding monomer N-acryloyl glycinamide (NAGA) mixed with polydopamine-coated gold nanorods (Au@PDA NRs). Then, the PNAGA-Au@PDA hydrogels were coated with *E. coli* or *S. aureus*-pretreated macrophage membrane (termed as E/SMM-PNAGA-Au@PDA). The hydrogel could identify and capture the target *E. coli* or *S. aureus*, which were killed by photothermal effects upon exposure to infrared radiation (Fig. 3G). Such strategies further demonstrate that the anti-infective value of CMCBs does not lie solely in the bactericidal activity of the core material, but also in the membrane-enabled enhancement of targeting, local retention, and biologically relevant pathogen interaction. However, its dependence on external NIR irradiation and bacteria-pretreated membranes may limit reproducibility and practical applicability in deep or irregular infected tissues.

Taken together, these anti-infective studies demonstrate that the key advantage of CMCBs lies not only in combining biomaterials with antimicrobial functions, but also in introducing membrane-mediated pathogen interception, toxin neutralization, and lesion-specific interaction. However, the current evidence remains largely proof-of-concept. Across the reported studies, common limitations include insufficient decoupling of membrane effects from core-material effects, limited evaluation in complex or mechanically dynamic tissue environments, and inadequate assessment of scalability, reproducibility, and long-term biosafety. These issues should be addressed to move anti-infective CMCBs closer to practical tissue-repair applications.

#### 3.1.2 Inflammatory targeting and microenvironmental regulation

Uncontrolled inflammation is a major barrier to effective tissue repair, not only because inflammatory mediators directly aggravate tissue injury, but also because prolonged or misdirected inflammatory responses impair the establishment of a regenerative microenvironment. Steroidal anti-inflammatory drugs (SAIDs) and nonsteroidal anti-inflammatory drugs (NSAIDs) are two common approaches for treating inflammatory diseases, and both are commonly used in clinical practice. However, in the absence of specific targeting, these drugs can lead to adverse side effects on the organism and exhibit poor therapeutic efficiency [85,86]. In this context, the value of CMCBs lies not merely in drug delivery, but in their ability to recognize inflammatory lesions through membrane-inherited receptors and adhesion molecules, thereby enabling localized intervention and, in some cases, subsequent modulation of the inflammatory immune niche. Compared with conventional anti-inflammatory therapies, which often suffer from poor targeting and systemic side effects, membrane camouflage offers a biomimetic strategy to improve lesion accumulation, prolong local action, and more precisely regulate pathological inflammation.

**Fig. 4:**
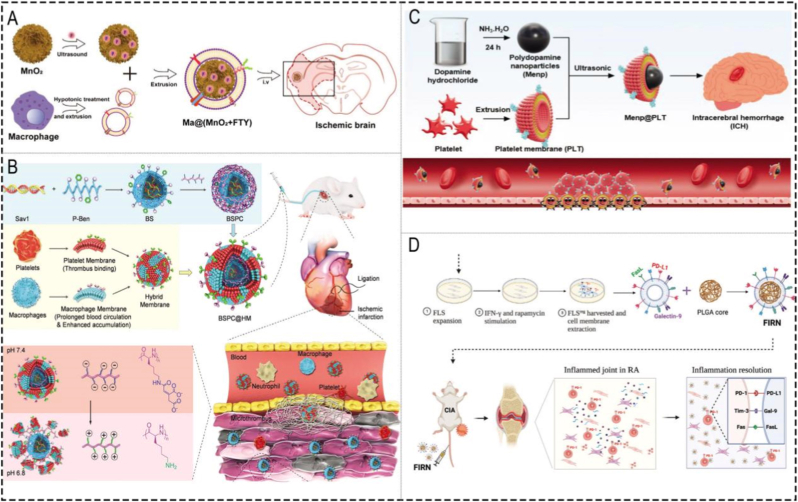
**(A)** Illustration of Ma@(MnO_2_+FTY) nanoparticles formation and treatment of damaged neurons in the ischemic brain [48]. **(B)** Schematic illustration of the biomimetic BSPC@HM NCs reversibly camouflaged with HM for the myocardial siSav1 delivery and IR injury management [45]. **(C)** Illustration of Menp@PLT nanoparticles formation and neuroprotection in ICH mouse 87. **(D)** Schematic illustration depicting the *in vitro* induction method for FLS^reg^ and the preparation and mechanisms of FIRN acting in RA treatment [88][].

This strategy has been explored in several acute inflammatory or ischemia-related injury models. In acute ischemic stroke, Li et al. found a macrophage-disguised honeycomb manganese dioxide (MnO_2_) nanosphere loaded with fingolimod (FTY) to improve the neuron inventory ratio of patients with acute ischemic stroke after reperfusion (Fig. 4A) [48]. Macrophage membrane could endow NPs for the precise targeting of inflammatory lesions, and MnO_2_ could catalyze H_2_O_2_ to O_2,_ which was generated after ischemia reperfusion, and FTY promoted the conversion of M1 macrophages toward the M2 phenotype, thereby reducing neuroinflammation and supporting neuronal repair. However, the respective contributions of lesion targeting, ROS modulation, and FTY-mediated immunoregulation were not fully separated, making it difficult to identify which component dominated the observed therapeutic benefit. Similarly, in another example of ischemia-reperfusion (IR) injury, Yin et al. developed a platelet-macrophage hybrid membrane reversible camouflage nanocomplex loaded with Sav1 siRNA for the treatment of myocardial ischemia-reperfusion injury (Fig. 4B) [45]. The combination of macrophage membranes with platelet membranes enhances the accumulation of damaged myocardial sites and innovatively overcomes the challenge of myocardial delivery, thus demonstrating the significance of hybrid membrane biomimetic technology. This study illustrates the added value of hybrid membrane biomimetics in highly complex lesions, where neither vascular injury recognition nor inflammatory targeting alone is sufficient.

Inflammatory targeting is also relevant in vascular injury-associated neurological disorders. Xu et al. illustrated the biological function of platelets targeting injured blood vessels and repairing injured blood vessels by designing platelet-membrane-modified polydopamine (Menp@PLT) (Fig. 4C) [87]. The platelet biomimetic materials in this work inherited proteins from platelets from top to bottom, imitating the role of platelets in brain hemorrhage through principles of biomimetics, which allowed them to target and accumulate at the site of vascular injury like natural platelets. Therefore, this novel strategy offers new insights and guidance for the therapy of bleeding disorders.

In chronic inflammatory disease, the role of membrane camouflage becomes even more closely linked to local immune regulation. Rheumatoid arthritis (RA) is characterized by persistent synovial inflammation of multiple joints, resulting in joint damage and involvement of vital organs. T cells in RA are continuously activated by synovial fibroblasts (FLS), posing a significant challenge for clinical therapy. Liu et al. designed a novel bionic treatment strategy for RA by coating IFN-γ and rapamycin-induced FLS^reg^-derived cell membranes on NPs (FIRN) (Fig. 4D). Compared with the positive control CIA mice receiving intravenous methotrexate (MRT) injection, FIRN treatment significantly reduced inflammatory cell infiltration and hyperplasia, preventing bone injury, and the therapeutic effect was much better than that of the MRT group. The relief of inflammation induced by the novel bionic treatment strategy facilitated the next step in bone tissue repair and regeneration [88]. However, the membrane source in this strategy is relatively specialized, which may limit scalability and reproducibility compared with more standardized membrane sources such as RBCs or macrophages.

**Fig. 5:**
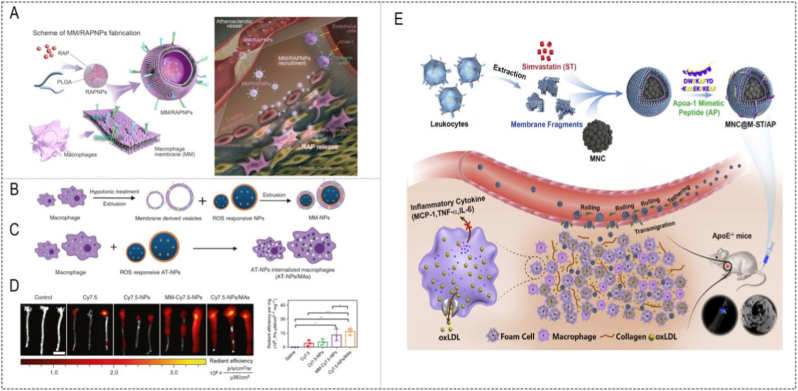
**(A)** Schematic of MM/RAPNP fabrication and its treatment for AS [22]. **(B)** Schematic illustration of the preparation of MM-NPs through an extrusion method [89]. **(C)** Schematic illustration of the preparation of AT-NPs/Mas. **(D)***Ex vivo f*luorescence bio-imaging and quantitative analysis of Cy7.5 fluorescent signal in aorta tissues. ApoE^−/−^mice fed with high-fat food for 1 month were i.v. administered with Cy7.5, Cy7.5-NPs, MM-Cy7.5-NPs, and Cy7.5-NPs/MAs, respectively [89][]. **(E)** Schematic illustration of the bioinspired MNC@M-ST/AP fabrication and its application for anti-AS by integrating multiple-targeting (ELVIS effect, natural inflammation-chemotaxis of leukocyte membrane and the targetable peptide AP), early detection (FL and MRI imaging) and dual-modality treatment (anti-inflammatory effect of ST and cholesterol-efflux promoting capacity of AP) [46].

Beyond these acute and autoimmune settings, membrane camouflage has also been widely explored in chronic vascular inflammatory lesions such as atherosclerosis. Wang et al. coated macrophage cell membrane onto the PLGA cores loaded with RAP (RAPNPs) to target and accumulate in atherosclerotic plaques to release anti-atherosclerotic drugs locally, thereby promoting the removal of atherosclerotic plaques, inhibiting the progression of AS, and promoting the tissue repair and regeneration of blood vessels (Fig. 5A) [22]. Similarly, Gao et al. developed a drug delivery nanoplatform for the treatment of AS, which could be described as macrophage membrane-coated ROS-responsive NPs (MM-NPs) (Fig. 5B) [89]. In this case, membrane camouflage not only improved lesion targeting but also provided a scavenging effect toward pro-inflammatory factors in the ROS-rich plaque microenvironment, thereby integrating targeting and microenvironment responsiveness (Fig. 5C/D). However, because rapamycin itself has potent anti-inflammatory and anti-proliferative activities, the membrane-specific contribution to plaque regression still requires more rigorous decoupling from the pharmacological effect of the payload[90][91]. Wu et al. developed self-driven biomimetic nanovesicles to achieve precise early AS management, in which the Fe_3_O_4_ magnetic nanoclusters (MNCs) were manufactured as a core for noninvasive magnetic resonance imaging (MRI), this biologically inspired free-riding strategies in white cell membranes will open new avenues for clinical application of biocompatible nano-systems for early AS detection and treatment (Fig. 5E) [46].

As a versatile and creative approach, CMCNPs integrate the merits of both synthetic nanomedicine and biological cell membrane systems. The complex components of original cell membranes, with the ability to target damaged tissues to achieve efficient tissue repair and regeneration, are provided by the "camouflaged" drug-delivery nanoplatforms developed *via* a top-down approach, which cannot be achieved with traditional chemical conjugation methods. Therefore, CMCNPs exhibit great potential for effective disease therapy and intervention. With the exploration of CMCNPs-related drug delivery nanoplatforms, more innovative nanotherapeutic drugs will be developed in the future to achieve functional optimization of biomimetic materials.

**Fig. 6:**
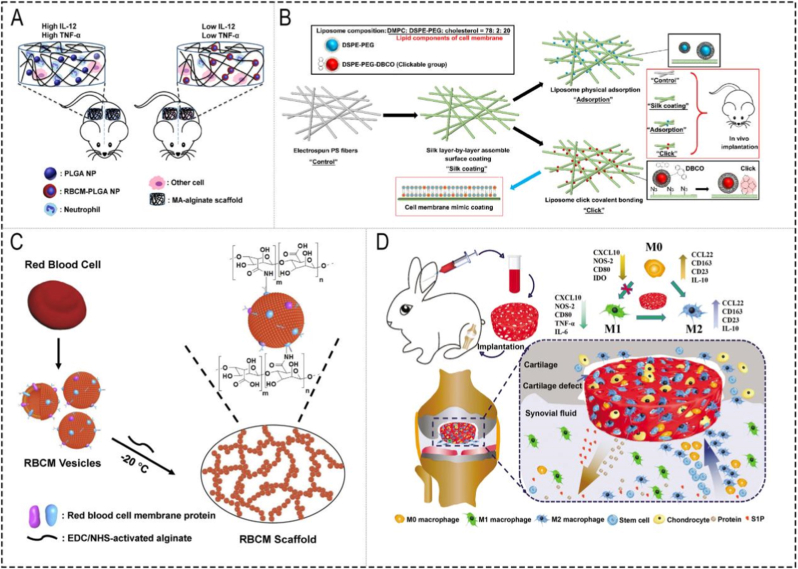
**(A)** Schematic illustration of the reduced inflammatory response to scaffold constructs after the coating of NPs with RBCM [24]. **(B)** Schematic illustration of liposome surface-modified electrospun polystyrene (PS) fibers *via* physical adsorption and click chemistry [93]. **(C)** Schematic illustration of macroporous RBCM scaffold and its fabrication [94]. **(D)** Schematic illustration of PLPMH scaffold preparation and endogenous M2 macrophage, M0 macrophage, and stem cell recruitment by releasing S1P and proteins for prompting cartilage defect repair [50].

Inflammatory targeting alone, however, is often insufficient to ensure successful tissue repair if the local immune microenvironment remains unfavorable. This is particularly relevant for implanted biomaterials, which frequently trigger foreign body responses (FBRs), neutrophil infiltration, and fibrous encapsulation [92]. Fan et al. showed that coating RBC membrane-coated PLGA nanoparticles into methacrylate alginate scaffolds could reduce neutrophil infiltration and eliminate the short-term inflammatory response to scaffold constructs (Fig. 6A) [24].This study represents the groundbreaking attempt to investigate the potential of cell membrane coating on nanoparticles in mitigating FBR, providing a prospective strategy for the application of cell-based scaffolds for tissue regeneration and immune regulation. In another study, Yang et al. developed a cell membrane-biomimetic coating, the bio-inert polystyrene (PS) was utilized as the model matrix for electrospinning, and then they deposited azide-bound silk fibroin on the fiber surface by layer-by-layer assembly, and finally liposomes were covalently modified using strain-promoted azide-alkyne cycloaddition (SPAAC) click chemistry to reduce FBR caused by the implantation of synthetic polymer scaffolds (Fig. 6B) [93]. In this study, a biomimetic cell membrane-like coating strategy was designed through the click-mediated fixation of liposomes followed by fusion onto the surface of electrospun fibers. Compared with previous physical coating, this biomimetic coating exhibited superior characteristics including enhanced homogeneity, fluidity, and notably, improved stability *in vitro* and *in vivo*. More importantly, this work presented a novel technique for surface modification of micro/nanofibers, offering versatile applications such as anti-FBR coatings and biomimetic substrates for cell membranes, which held potential in diverse fields including tissue repair patches, postoperative anti-adhesion barriers, and drug delivery systems *in situ*.

Based on the immunomodulatory performance of cell membrane vesicles and the characteristics of hydrophobic drugs carried by scaffold materials [29], Fan et al. first reported a new type of scaffold named RBCM-derived porous hydrogel scaffolds that has excellent biocompatibility and can influence the cell activity, compared with MA-alginate scaffolds, the RBCM scaffolds recruited more M2 macrophages *in vivo*, and phosphatidylserine in the membrane vesicles appeared to promote macrophage polarization toward an anti-inflammatory phenotype (Fig. 6C) [94]. This study is particularly informative because it connects membrane composition with a specific immunoregulatory mechanism rather than only reporting a phenomenological anti-inflammatory effect. There will be wider application of this type of CMMBSs in tolerance induction and excessive inflammation, though the specific interactions between distinct vesicles and recruited cells are expected to be determined in tissue repair [95]. However, the precise interactions between distinct membrane components and recruited immune cells remain incompletely defined, which limits mechanistic generalization to other scaffold systems.

A similar idea has also been applied to osteoarthritis-associated inflammatory degeneration. Because persistent synovial inflammation and macrophage imbalance can exacerbate chondrocyte apoptosis and cartilage degradation, long-term microenvironmental regulation is essential for cartilage repair. Pan et al. developed a PRP macroporous hydrogel (PLPMH) scaffold for cartilage repair (Fig. 6D) [50], which was an excellent platelet-derived biomaterial, showing potential in effectively recruiting M2 macrophages and maintaining a long-term anti-inflammatory microenvironment to promote cartilage regeneration by virtue of PRP's continuous release of bioactive substances and the macroporous structure. However, because PRP-derived systems contain highly heterogeneous bioactive factors, their batch consistency and mechanism-specific reproducibility may be more difficult to control than those of more defined membrane-camouflaged platforms.

Taken together, these studies suggest that the therapeutic contribution of CMCBs in inflammatory diseases should not be viewed solely as “targeting inflammation.” Rather, their real advantage lies in coupling membrane-mediated lesion recognition with localized intervention and, when properly designed, subsequent regulation of the inflammatory microenvironment. At the same time, the current literature remains largely proof-of-concept. Common limitations include insufficient separation of membrane effects from core-material or drug effects, incomplete mechanistic clarification of receptor-mediated targeting, and limited evaluation of long-term integration in complex tissue environments. Addressing these issues will be essential for moving CMCBs from lesion-targeting nanoplatforms toward truly regenerative biomaterial systems.

### 3.2 CMCBs for regenerative remodeling and functional restoration

Beyond controlling infection and inflammatory microenvironments, another major contribution of CMCBs to tissue repair lies in their ability to support regenerative remodeling and functional restoration. By integrating the structural advantages of biomaterial carriers with membrane-derived biointerfaces, CMCBs can enhance cell adhesion, survival, proliferation, angiogenesis, and tissue-specific repair, while in some pathological settings also attenuating excessive matrix deposition, fibrosis, and scar formation. Accordingly, the following section focuses on two interconnected aspects of this regenerative role: the promotion of regenerative cell bioactivity and the attenuation of fibrosis-related remodeling.

#### 3.2.1 Promotion of regenerative cell bioactivity

Effective tissue regeneration requires not only the resolution of injury-associated inflammation, but also the establishment of a permissive microenvironment that supports cell adhesion, proliferation, migration, and functional activation. In this context, biomaterial scaffolds have long been explored as structural platforms for tissue repair. However, conventional scaffolds are often limited by insufficient biomimicry and a lack of specific biological cues for resident or infiltrating cells. Cell membrane camouflage provides a useful strategy to overcome these limitations, because membrane-derived interfaces can present endogenous surface molecules and microenvironmental signals while preserving the structural support of the carrier material. Therefore, CMCBs can promote regenerative cell bioactivity not simply by acting as passive scaffolds, but by providing both topographical support and cell-mimetic biofunctional cues.

**Fig. 7:**
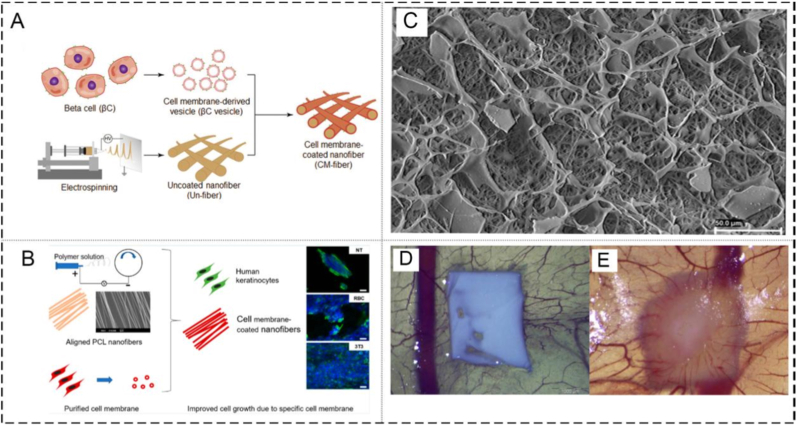
**(A)** Preparation and characterization of CM-fibers[23].**(B)** Schematic illustration of PRP-PCL scaffolds preparation [53]. **(C)** SEM microphotographs of PRP-PCL scaffolds [97]. **(D)** and **(E)** Effect of PRP-PCL scaffolds on CAM angiogenesis at day 12 of incubation of fertilized hen eggs. [97]

Cell membrane-camouflaged scaffolds can provide both a three-dimensional structure for cell adhesion and proliferation and physicochemical or bioactive cues for resident cells. Chen et al. successfully prepared cell membrane-coated nanofibers (CM-fibers) by collecting beta-cell membrane-derived vesicles and then coating them onto polymeric nanofibers. The study verified the feasibility of CM-fibers as scaffolds for the improvement of cell growth and bioactivity (Fig. 7A) [23]. Although the CM-fibers could present both cell surface antigens and topographic clues for tissue regeneration, it is still unclear whether the change of cell behavior on cell membrane-modified scaffolds is influenced by specific interactions between cells. To apply the platform to other types of cells and tissues, Wang et al. prepared fibroblast membrane-coated electrospun fiber scaffolds, and verified that the scaffolds promoted the growth of keratinocytes through cell type-specific interaction (Fig. 7B) [53]. When cells are cultured in these modified scaffolds, the cell membrane provides a natural environment and bio-functionality of cell membrane-coated scaffolds, and the specific cell−cell interactions are preserved to modulate cellular response, which promotes interactions between cells similar to those in normal tissues, resulting in better cell survival and performance. Poly(ε-caprolactone) (PCL), with no risk of creating a detrimental acidic milieu for cell growth, is considered a biocompatible and biodegradable polymer. Nevertheless, the hydrophobic property and bio-inertness of PCL scaffolds restrict mammalian cell attachment, which could be reversed by hydrophilic techniques such as wet chemistry, plasma treatment, surface graft polymerization, or physisorption. PRP harbors a plethora of bioactive factors crucial for promoting tissue repair. Furthermore, the above techniques not only facilitate the integration of PRP but also enhance cell adhesion [51,96]. Luis et al. successfully prepared bioactive PCL scaffolds (PRP-PCL scaffolds) by coating a layer of PRP on the surface of electrospun fibers via impregnation and freezing. An obvious pro-angiogenic effect of PRP-PCL scaffolds on the allantoic vessels could be observed (Fig. 7C) [97]. In addition, PRP significantly improved the encapsulation of the PCL scaffold in the chicken chorioallantoic membrane (Fig. 7D-E). Therefore, this work demonstrates the ability of the PRP-PCL scaffolds to maintain the release of certain growth factors and its role as a tissue engineering platform to promote cell apposition, proliferation, and CAM angiogenesis [51]. However, this study mainly demonstrated short-term pro-angiogenic and proliferative effects, whereas the durability and tissue specificity of these benefits in clinically relevant defect models remain uncertain.

Taken together, these studies indicate that the regenerative value of CMCBs in this context lies in their ability to convert biomaterial scaffolds from passive structural substrates into biointeractive platforms that support cell adhesion, survival, proliferation, and angiogenesis. At the same time, current evidence remains largely proof-of-concept, and future studies should more clearly distinguish membrane-specific biological effects from those attributable to scaffold architecture, released growth factors, or general improvements in surface compatibility.

#### 3.2.2 Attenuation of fibrosis and scar formation

Successful tissue repair depends not only on regenerative stimulation, but also on preventing maladaptive remodeling. When inflammation-mediated matrix remodeling becomes dysregulated, excessive extracellular matrix deposition, persistent fibroblast activation, and abnormal vascular responses can lead to fibrosis and scar formation rather than functional tissue restoration. In this setting, the therapeutic significance of CMCBs lies in their ability to regulate inflammatory and oxidative microenvironments while simultaneously modulating cell behavior related to repair and remodeling. Compared with conventional antifibrotic approaches, CMCBs may improve lesion targeting and local bioactivity, thereby offering a more integrated means of limiting pathological fibrosis when implanted in damaged tissues.

**Fig. 8:**
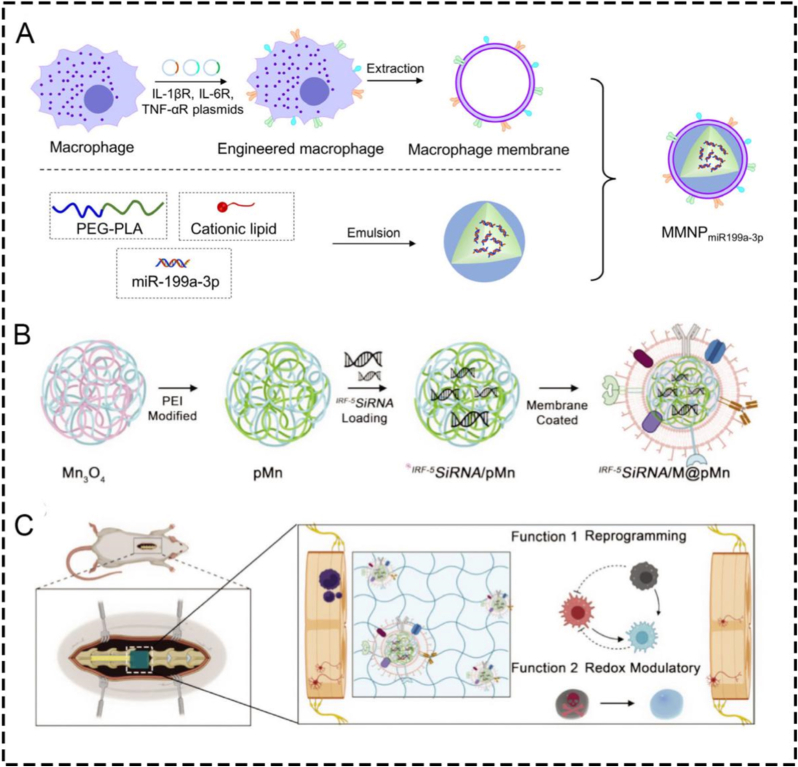
**(A)** Schematic representation of engineered macrophage membrane-enveloped nanoparticles encapsulating miR199a-3p [49]. **(B)** Schematic representation of integrated IRF-5SiRNA/M@pMn nanozymes. Polyethyleneimine (PEI) was used to modify Mn_3_O_4_ to form pMn. **(C)** Schematic diagram of the multifunctional therapeutic ability of the prepared nanozymes that play the main function [52]**.**

Myocardial infarction (MI) is one of the most serious ischemic diseases that can lead to heart failure. Xue et al. prepared macrophage membrane-coated nanoparticles (MMNPs) containing miR199a-3p to address myocardial cell apoptosis, inflammation, and fibrosis in the pathogenesis of MI [49]. The encapsulation of macrophage membranes significantly promoted the cardiovascular cells' efficient uptake of TNF-α, IL-1, and IL-6 receptors, which were overexpressed on MMNPs, thus inhibiting the inflammatory response (Fig. 8A), and significantly decreasing the myocardial fibrotic area. Therefore, MMNPs improved left ventricular remodeling and cardiac function and prevented MI, suggesting that mir199a-3p-containing MMNPs are a potential therapeutic approach for MI. However, the respective roles of macrophage membrane targeting and miR199a-3p-mediated intracellular regulation were not fully decoupled, making the dominant antifibrotic mechanism difficult to define.

A related strategy has been reported for spinal cord injury (SCI), where nerve survival and regeneration at the injured site are difficult due to the dynamic and complex injury microenvironment. Oxidative stress and inflammation predominate after external nerve injury. Xiong et al. designed a combination of novel "nanoflower" Mn_3_O_4_ integrated with "pollen" ^*IRF−5*^*SiRNA* wrapped with neutrophil (Fig. 8B) [52]. Interestingly, "pollen" ^*IRF−5*^*SiRNA* can reverse the phenotype of inflammatory macrophages by reducing the expression of interferon regulatory factor 5 (IRF-5). Neutrophil membrane coating integrates nanozymes to further protect and target the delivery of "pollen" ^*IRF−5*^*SiRNA* to inflammatory macrophages, thereby effectively reducing inflammatory cell infiltration and thereby reducing nerve scarring (Fig. 8C). By integrating siRNA technology with the development of multifunctional nanozymes, a synergistic approach for combating inflammation and oxidative stress in spinal cord injury is realized. The strategy of this work could be applied to a range of disease models, including the neurological, immunological systems, cardiovascular, and aging. However, the therapeutic benefit of this platform may depend strongly on the synchronization of targeting, antioxidative activity, and gene silencing, which could be difficult to control across different injury stages.

Overall, the studies in this subsection indicate that CMCBs can contribute to antifibrotic repair not only by suppressing excessive inflammation, but also by reshaping the remodeling milieu in a way that limits pathological matrix deposition and preserves functional recovery (Table 2). Nevertheless, most current studies remain at the stage of mechanistically promising proof-of-concept demonstrations. Greater emphasis should be placed on long-term functional evaluation, decoupling membrane effects from core-material or cargo effects, and assessing whether these antifibrotic benefits can be reproduced in mechanically dynamic and clinically relevant tissue environments.

## 4 Exosome-modified biomaterials (EMBs)

Exosomes, as phospholipid bilayer vesicles naturally secreted by cells that are always taken up by recipient cells through autocrine or paracrine secretion from the cell and affect the functional status of other cells, can serve as carriers to transmit biological information and regulate cell behavior [35,98,99]. Exosomes, containing a variety of biomacromolecules such as proteins, lipids, and nucleic acids, have been demonstrated to be traced to the source cells but differ in terms of content ratio [100]. Exosomes have shown great potential in tissue repair and regeneration for their satisfactory application in angiogenesis, immune modulation, reduction of apoptosis, and spectrum-specific differentiation [101]. Nonetheless, after the production through donor induction, somatic cell induction, or direct exosome induction, exosomes may be cleared before reaching the target tissue due to a deficiency of regulatory release mechanisms, nanoscale size, or poor bioavailability [102]. Therefore, membrane fusion, physical insertion, protein modification, or gene editing is significant for the enrichment of exosomes in appropriate lesions [103]. Mounting evidence has justified the feasibility of the 3-D structure and interconnected pores in the hydrogel, nanofiber, nano-tube, or scaffolds on the regulation of exosome embedding accommodation, exosome loss reduction and integrity of proteins and microRNAs (miRNAs). Unlike CMCBs, which primarily endow biomaterials with biomimetic interfacial properties through membrane camouflage, EMBs are designed to use biomaterials as carriers for the localization, protection, and controlled release of exosomes. Therefore, the therapeutic performance of EMBs depends not only on the intrinsic bioactivity of exosomes but also on whether the combined biomaterial can preserve vesicle integrity, prolong local retention, and match exosome release with the temporal demands of tissue repair. In this section, the roles of EMBs are discussed under two closely related aspects: facilitating repair and reducing apoptosis.

### 4.1 Facilitating repair

As mentioned before, the process of cell proliferation, migration and differentiation plays a significant role in tissue repair [101]. A variety of studies have exhibited that exosomes participate in these processes mainly in two ways. On the one hand, growth factors and chemokines such as VEGF, epidermal growth factor, and S1P, *etc.* contained in exosomes can promote the proliferation and migration of target cells, which is helpful for the accumulation of seed cells locally [104,105]. On the other hand, as the essential carriers of cell-cell communication, exosomes can deliver informatic molecules into target cells to activate downstream signaling pathways, achieving the specific differentiation of stem cells [106].

**Fig. 9:**
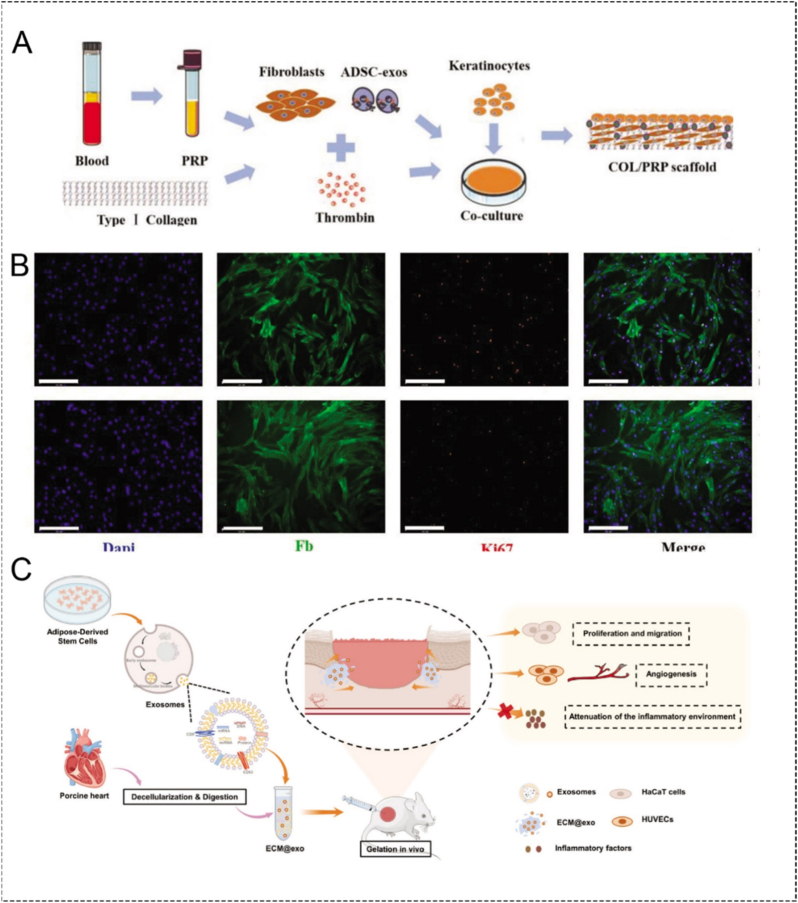
**(A)** Scheme of the preparation of the exosome/cell-loaded COL/PRP scaffold [54]. **(B)** The effect of the exosomes on fibroblast proliferation was determined using Ki67 immunofluorescence [54]. **(C)** Scheme of pH-responsive exosomes releases in FHE hydrogel[55].

Compared with direct exosome injection, the incorporation of exosomes into hydrogels, scaffolds, or adhesive patches can better maintain local bioavailability and improve the spatiotemporal coordination between exosomal signals and tissue repair. For example, Wang et al. developed adipose-derived mesenchymal stem cell-derived exosomes with type I collagen/platelet-rich plasma scaffolds (COL/PRP scaffolds) for wound healing (Fig. 9A) [54]. In the immunofluorescence image shown in Fig. 9B, the fibroblasts co-cultured with ADSC-exos on the scaffolds have higher expression of Ki67 (a proliferation-related marker). Similarly, Wang et al. designed an encapsulated adipose-derived mesenchymal stem cell-derived exosomes into an ECM-derived hydrogel for chronic wound healing (Fig. 9C) [55]. The results showed that continuously released AMSCs-exos promoted the proliferation of human umbilical vein endothelial cells (HUVECs), thereby promoting wound healing. The ability of exosomes to promote local cell aggregation and differentiation can accelerate the process of tissue repair, and the mechanism of exosomes changing cell behavior should be further explored to achieve more diverse cell regeneration, such as the regeneration of glandular cells, neurons or local monopotent stem cells. Meanwhile, the excellent physical and chemical properties of biomaterials can achieve precise bionics of body tissues. If the precise loading of exosomes inside biomaterials can be achieved on this basis, it is expected to achieve more sophisticated functional reconstruction of tissues.

Usually, cell proliferation is accompanied by cell migration. ECM provides structural and functional support for cell migration. In addition, miRNAs and proteins in exosomes influence cell motility, by which exosomes promote directional migration of cells around damaged tissues to areas requiring repair and accelerate tissue repair and regeneration. Letizia Ferroni et al. developed exosomes-functionalized methacrylated hyaluronic acid (MeHA) to prepare wound patches through 3D bioprinting technology [107]. In this work, hMSC-Exos can effectively penetrate into MeHA and promote the proliferation and migration of fibroblasts, thereby facilitating wound healing. Zhao et al. studied the impact of hAEC-Exos combined with PROse or RNase A on fibroblast and skin wound healing. The experiments *in vitro* and *in vivo* demonstrated that miRNAs derived from hAEC-Exos can enhance fibroblast growth and movement, leading to faster wound healing. This highlights the crucial role of exosomal miRNAs in the healing process [108]. More specifically, increasing evidence suggests that the regenerative effects of EMBs may depend on defined exosomal cargos rather than on undefined “bioactive factors” in general. For instance, exosomal miR-130b-5p has been shown to promote wound healing by targeting TGFBR3, indicating that biomaterial-assisted exosome delivery may work through preserving and releasing specific pro-regenerative miRNA programs.

Taken together, these studies indicate that EMBs facilitate repair not simply because exosomes are “bioactive,” but because biomaterials can localize, protect, and gradually release exosomal cargos in a way that better matches the requirements of tissue regeneration. At the same time, current studies still often stop at morphological or short-term repair outcomes, and greater emphasis should be placed on identifying cargo-specific mechanisms, clarifying the contribution of the biomaterial carrier itself, and determining whether structural improvement can truly translate into durable functional restoration.

### 4.2 Inhibiting apoptosis and necrosis

When the organism is injured, the occurrence rate of apoptosis or necrosis increases significantly due to peroxidation or abnormal inflammation. Antioxidant enzymes in exosomes can reduce intracellular oxidative stress and protect cells from damage. Moreover, exosomes play an immunomodulatory role by reducing the expression of macrophage chemotactic protein CX3CL1 and TNF, up-regulating IL-10, and reducing the local inflammatory response, thus promoting tissue repair [109,110]. Exosomes can regulate oxidative stress after injury, reducing local ROS levels and minimizing damage to surrounding cells, ultimately inhibiting apoptosis. MSC-Exos, derived from mesenchymal stem cells, have been found to contain high levels of glutathione peroxidase. This enzyme has the ability to inhibit oxidative stress and apoptosis caused by hydrogen peroxide and carbon tetrachloride in liver cells [98]. Studies have also shown that intravenous injections of MSC-Exos can reduce inflammatory cell infiltration, decrease the release of inflammatory cytokines, alleviate oxidative stress, and ultimately reduce hepatocyte apoptosis, necrosis, and liver injury in a hepatic ischemia-reperfusion model [111].

**Fig. 10:**
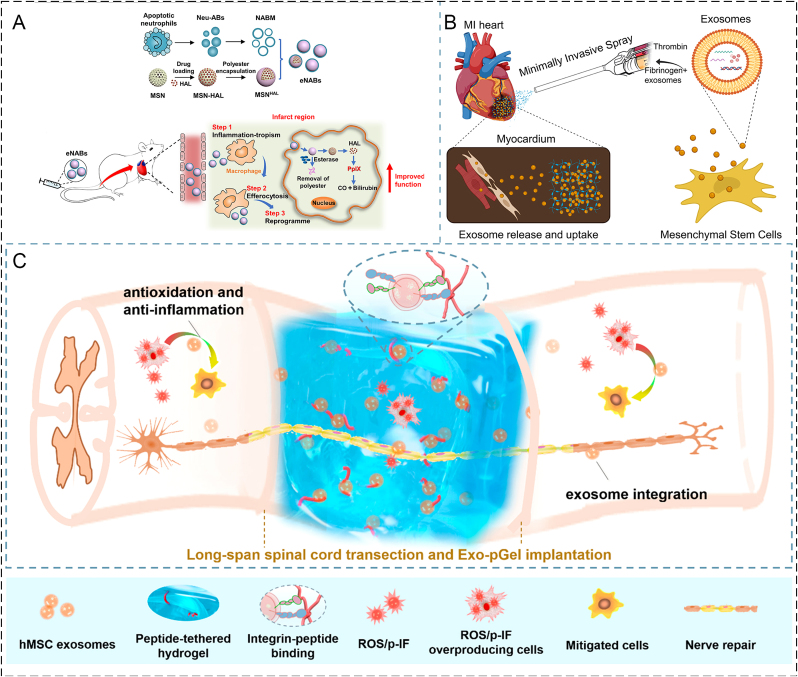
**(A)** Synthetic scheme of the eNABs [56]. **(B)** Application diagram of a minimally invasive spray loading with bone marrow MSC-derived exosomes to form *in situ* fiber patches. The exosomes can promote the proliferation of cardiac vessels and muscle after myocardial infarction in a safe and effective way [57]. **(C)** The component and application of the Exo-pGel reduce the local inflammation level to promote the functional repair of the spinal cord [58].

The incidence of immune reactions and inflammation also tends to increase significantly at the injury site. Exosomes can reduce inflammation by inhibiting the production and release of inflammatory factors and regulating the differentiation and polarization of immune cells, which in turn reduces the apoptosis of tissue cells. After myocardial infarction, infiltration of a large number of neutrophils and other inflammatory cells leads to delayed regression of inflammation, hindering tissue repair and leading to impaired cardiac function. Under physiological conditions, by releasing extracellular vesicles (EVs) such as apoptotic bodies that inherit signal molecules from the cell surface, apoptotic neutrophils promote intercellular communication, thus initiating macrophage polarization and inflammatory regression. Bao et al. developed engineered neutrophil apoptotic bodies (eNABs) by fusing the natural membrane of neutrophil apoptotic bodies with mesoporous silica nanoparticles (MSN^HAL^) loaded with hexyl 5-aminolevulinate (HAL) (Fig. 10A) [56]. This combination approach of eNABs and HAL effectively reprograms infarct macrophages, regulates the inflammatory response, and promotes tissue regeneration. This broad rationale supports the use of EMBs in neurologic and ischemic tissues, but it also underscores the need to move beyond descriptive claims and identify which exosomal cargos are actually responsible for cytoprotection in each disease context. Exosomes can also inhibit apoptosis through gene regulation. MI, which is currently one of the leading causes of death worldwide, has been confirmed about its main pathogenesis as hypoxia-induced oxidative stress [112,113]. Exosomes play an important role in resistance to myocardial infarction and post-infarction repair [114]. For example, MSCs-derived exosomes can target apoptotic genes and reduce apoptosis levels, and MSCs-derived exosomes from human umbilical cord blood can promote the differentiation of fibroblasts into myofibroblasts in the post-infarction environment and inhibit myocardial remodeling [34,115,116]. However, the application of exosomes in cardiac repair is greatly limited by exosome retention in the heart. From this perspective, Yao et al. developed a spray consisting of a mixture of bone marrow MSC-derived exosomes and fibrinogen with a thrombin solution, which was sprayed at the heart to form a fibrin patch *in situ* (Fig. 10B) [57]. This spray increased the retention and uptake of exosomes in the heart, leading to a reduction in cardiomyocyte apoptosis. It was also effective in reducing the size of myocardial infarction in a mouse model. Similarly, the Exo-pGel system, which integrates human MSC-derived exosomes with an adhesive hydrogel, was reported to reduce local inflammation and promote the functional repair of the spinal cord (Fig. 10C) [58]. Researchers have indicated that EVs can enhance neuronal survival and regeneration by delivering neuroprotective factors and anti-apoptotic molecules, presenting novel strategies for diseases like Parkinson's disease, Alzheimer's disease, and spinal cord injuries. With the assistance of different forms of biomaterials, exosomes can effectively act on complex anatomical structures to achieve functional reconstruction of tissues.

More mechanistic evidence from the broader exosome field indicates that exosomal miRNAs can be key mediators of anti-apoptotic repair [117]. For example, MSC-derived exosomal miR-21a-5p has been shown to exert cardioprotective effects by downregulating PDCD4, PTEN, FasL, and Peli1. In contrast, hUCMSC-derived exosomal miR-146b can protect neurons in spinal cord injury by targeting TLR4 and suppressing NF-κB signaling. These examples suggest that EMBs should be interpreted not only as exosome-retaining biomaterials, but also as platforms for preserving and delivering defined anti-apoptotic and anti-inflammatory miRNA programs. Therefore, the anti-apoptotic role of EMBs should be understood from a biomaterials-centered perspective: the biomaterial is not a passive vehicle, but a regulator of whether exosomes can remain active, persist long enough in the lesion, and release their cargos during the critical period when salvageable cells are still present. Nevertheless, many current studies still primarily demonstrate reduced ROS, lower levels of apoptosis markers, or improved histology, without fully clarifying cargo-level mechanisms or demonstrating long-term functional integration *in vivo*(Table 3). Future studies should therefore pay greater attention to exosome heterogeneity, cargo preservation after loading, and the relationship between release kinetics and durable tissue recovery.

## 5 Challenges and perspectives

Biomaterials provide a structural framework that facilitates the attachment and migration of host stem and progenitor cells, thus achieving tissue repair and regeneration. However, this process highly relies on the precise control of (bio)physiochemical cues to direct endogenous cells to the site of injury. In addition, CDVMBs must also satisfy stringent quality requirements to ensure efficacy and safety for clinical application. At this stage, the major bottlenecks of CDVMBs are no longer limited to proof-of-concept therapeutic efficacy but increasingly involve controllable manufacturing, vesicle integrity, dynamic therapeutic programming, and clinical translation. Moreover, these bottlenecks differ between CMCBs and EMBs: CMCBs require preservation of membrane topology, receptor orientation, and coating homogeneity, whereas EMBs require control over extracellular vesicle identity, potency, batch consistency, and release behavior after incorporation into biomaterial carriers [118].

(1) **Large-scale production and high reproducibility**: For CMCBs, the main bottleneck is not simply the demand for large numbers of source cells, but the difficulty of obtaining membrane fractions with preserved surface proteins, low contamination, and reproducible coating quality. Different membrane extraction and coating procedures, such as extrusion, sonication, and electrostatic self-assembly, may affect membrane integrity, coating completeness, and receptor exposure, thereby influencing biological performance. For EMBs, vesicle isolation efficiency remains a major challenge, as conventional ultracentrifugation is labor-intensive, low-throughput, and operator-dependent. In contrast, precipitation-based methods often increase yield at the expense of purity. Although tangential flow filtration, hollow-fiber bioreactors, and microfluidic platforms are emerging as more scalable alternatives, they still require careful balancing of yield, purity, sterility, and potency. Therefore, future scale-up should move toward closed, automated, and GMP-compatible workflows with standardized in-process quality control [119].
(2) **Long-term storage:** Long-term storage is limited not only by the denaturation of membrane proteins, but also by vesicle aggregation, fusion, cargo leakage, adsorption to containers, and repeated freeze–thaw injury. For EV-containing formulations, storage in plain PBS is often insufficient to preserve particle recovery, surface-marker expression, RNA integrity, and cellular uptake activity over time, whereas stabilizing buffers and lyophilization strategies with protectants such as trehalose and albumin appear more favorable. In addition, CDVMBs face a “post-formulation stability” problem: vesicles may remain stable in suspension but lose activity after coating nanoparticles or after embedding in hydrogels or scaffolds. Therefore, stability assessment should include not only particle retention, but also receptor bioactivity, exosomal potency, release kinetics after reconstitution, and robustness during transport and storage.
(3) **Biophysical integrity and standardization: membrane orientation and exosome heterogeneity:** For CMCBs, membrane orientation is a critical yet often overlooked engineering parameter. If the membrane is coated inside-out rather than right-side-out, extracellular receptors and glycans are not properly exposed, thereby compromising immune evasion, lesion recognition, and interfacial signaling. Thus, “successful coating” should not be judged solely by changes in particle size or zeta potential; it should also include assessment of sidedness, fluidity, protein retention, and coating completeness using orthogonal techniques, such as immunogold TEM, sidedness-sensitive flow cytometry, and functional receptor assays. For EMBs, batch-to-batch heterogeneity is equally important. Even exosomes produced by a single cell type contain subpopulations with different sizes, membrane marker profiles, cargo compositions, biodistribution patterns, and biological potency. Donor variability, cell senescence, culture conditions, stimulation methods, and purification strategies further amplify this heterogeneity. Therefore, future EMB development should incorporate single-vesicle or multi-parametric characterization, together with standardized potency assays linked to the intended mechanism of action, rather than relying only on bulk particle counts or canonical EV markers.
(4) **Biosafety, preclinical validation, and clinical translation:** Although CDVMBs have shown encouraging therapeutic effects in wound healing, myocardial repair, spinal cord injury, bone regeneration, and other models, most studies still rely on small-animal experiments and short-term efficacy readouts. More translationally relevant evaluation is needed, including biodistribution, persistence, off-target accumulation, immunogenicity, thrombogenicity, tumorigenicity, and degradation behavior *in vivo.* In particular, the biological safety of biomaterial-integrated vesicles cannot be inferred directly from free vesicles alone, because biomaterials may alter local retention, immune exposure, and clearance kinetics. For allogeneic membrane sources, residual intracellular contents, donor-derived antigens, and adventitious agents must be strictly controlled. Therefore, future preclinical studies should incorporate disease-relevant large-animal models, repeated-dose studies, route-specific toxicology, and multimodal tracking of vesicle fate and host responses. Importantly, the field is already moving beyond purely conceptual work: EV therapeutics have entered early clinical translation, including first-in-human platelet-derived EV studies for wound healing and GMP-manufactured EV-enriched secretome products approved for phase I cardiac trials. These advances suggest that clinically testable CDVMB-like formulations are feasible, but only when potency, safety, and manufacturing are defined more rigorously.
(5) **Dynamic and stimuli-responsive CDVMBs for stage-specific healing:** Tissue repair is an intrinsically dynamic and multi-stage process. In the early stage, antibacterial activity and inflammatory control are often dominant, whereas angiogenesis, matrix deposition, stem/progenitor activation, and functional remodeling become more important at later stages. Therefore, next-generation CDVMBs should evolve from passive vesicle carriers into programmable systems that respond to local or external cues. For example, pH-, ROS-, glucose-, and enzyme-triggered matrices may enable lesion-adaptive release, whereas near-infrared-, magnetic-, or ultrasound-responsive systems may enable on-demand activation at specific healing stages. Such strategies are particularly relevant for chronic wounds, infected bone defects, and ischemic tissues, where the pathological microenvironment changes over time. In this context, appropriate inflammation management and controllable macrophage polarization should be interpreted not only as “which phenotype is beneficial”, but also as “when, where, and to what degree should vesicle signals be released” [120].
(6) **Regulatory classification and GMP translation:** Clinical translation of CDVMBs will also depend on how they are classified and manufactured. From a regulatory perspective, CDVMBs will likely require case-by-case evaluation according to source material, degree of manipulation, mechanism of action, and whether the biomaterial component contributes directly to therapeutic function. In the United States, exosome products intended to treat human diseases are regulated as drugs/biological products, and there are currently no FDA-approved exosome products. In addition, if a vesicle component is integrated with a scaffold or synthetic carrier that performs a device-like role, the product may also raise combination-product considerations. In Europe, vesicle-containing regenerative products may require advanced-therapy-related classification or other advanced medicinal-product evaluation depending on their composition and intended use. Accordingly, GMP translation requires more than high yield; it requires traceable donor or cell banks, controlled culture systems, closed or aseptic downstream processing, validated sterility strategies, release assays for identity, purity, potency, and safety, as well as stability programs that support transport and shelf life. Recent GMP studies have demonstrated that EV-enriched secretome products can be manufactured using scaled-up workflows with release testing, toxicity assessment, and low-temperature stability, providing an important translational reference for future CDVMB development.
(7) **Future roadmap:** Over the next 5–10 years, CDVMB research is likely to shift from empirical biomimicry toward data-driven and mechanism-guided engineering. First, AI-assisted vesicle engineering may help predict optimal membrane sources, cargo compositions, scaffold parameters, and release profiles, thereby reducing trial-and-error design. Second, programmable biomimetic materials, including shape-adaptive and 4D-printed scaffolds, may allow vesicle presentation and therapeutic output to evolve with mechanical loading, swelling, degradation, or inflammatory cues. Third, microfluidic fabrication platforms are expected to improve vesicle production, purification, coating homogeneity, and on-chip quality control. Finally, spatial transcriptomics, single-vesicle profiling, and multimodal *in vivo* imaging may provide a more precise map of vesicle fate, tissue distribution, and cell-specific regenerative responses in living tissues. Taken together, the future of CDVMBs lies not simply in combining vesicles and materials but in building intelligent regenerative systems with controllable topology, defined potency, adaptive release, and clinically compatible manufacturing.

## 6 Conclusions

In this review, mechanisms of tissue repair and regeneration as well as imbalanced regulation during the repair process were analyzed, and a strategy of CDVMBs to promote tissue regeneration by regulating the "repair balance" was proposed. With the natural properties of cell-derived membranes to interact with the microenvironment, CDVMBs offer new solutions for managing tissue regeneration. On the one hand, biomaterials can provide a solid matrix to support cell adhesion, penetration, and proliferation, providing a framework and components for tissue repair and regeneration. On the other hand, cell membrane vesicles further endow CDVMBs with unparalleled biointerface performance to achieve inflammatory factors neutralization, lesion site targeting, and pathogen removal. Moreover, bioactive molecules released from exosomes can be delivered to damaged sites to facilitate tissue remodeling and regeneration. Interestingly, cell-derived vesicles and biomaterials even coordinate in immune regulation, providing new therapeutic strategies and methods. Therefore, CDVMBs show great potential as a promising approach for tissue repair and regeneration.

CMCBs and EMBs, as two potential modification strategies of biomaterials, were mainly described in this review. CMCBs not only control infection and alleviate local excessive inflammatory responses but also interact with surrounding tissues by coupling with biomaterials to provide a macroscopic, active environment for tissue repair, exhibiting excellent characteristics for regulating inflammation and promoting remodeling. EMBs play an indispensable role in cell regulation during the repair process, promoting cell proliferation and inhibiting cell apoptosis to facilitate tissue repair and regeneration, which also contributes to functional reconstruction. By integrating exosomes with biomaterials, not only can the biological signals within exosomes be utilized to regulate the repair microenvironment, but different biomaterials can also enhance their mechanical and chemical properties, which better facilitate tissue repair and regeneration.

Although the great application potential of CDVMBs has been exhibited, the therapy of CDVMBs has not yet entered the clinical trial stage due to the limitations of stability, safety, effectiveness, and large-scale production. Fortunately, these foreseeable challenges can be overcome through material safety improvements and policy adjustments. In the context of application development, more in-depth mechanisms of material-cell interaction will be elaborated.

## Consent for publication

All authors have approved the final draft of this manuscript for submission and have given consent for the publication of identifiable details.

## Ethics approval and consent to participate

Not applicable.

## CRediT authorship contribution statement

**Wanli Song:** Conceptualization, Investigation, Writing – original draft. **Junmiao Xue:** Conceptualization, Investigation. **Pengfei Jia:** Resources, Software, Visualization. **Yongzhi Xu:** Data curation, Software. **Hao Jiang:** Formal analysis, Writing – original draft. **Zhiyu Yuan:** Visualization, Writing – original draft. **Tianyi Zhang:** Methodology, Writing – original draft. **Wenping Zhang:** Methodology, Writing – original draft. **Jingyang Zhao:** Conceptualization, Resources, Writing – review & editing. **Xiaohui Qiu:** Conceptualization, Funding acquisition, Writing – review & editing. **Qihui Zhou:** Conceptualization, Funding acquisition, Project administration, Resources, Writing – review & editing.

## Declaration of competing interest

The authors declare that they have no known competing financial interests or personal relationships that could have appeared to influence the work reported in this paper.

## Data Availability

No data was used for the research described in the article.
